# Genomic characterization of *bla*_IMP_-harboring plasmids in *Klebsiella* spp.

**DOI:** 10.1128/spectrum.03790-25

**Published:** 2026-07-02

**Authors:** Mingxiao Chen, Tingting Deng, Jingyi Zhang, Yitong Han, Jingjie Li, Minling Wang, Yuqing Weng, Yu Gan, Xiaobin Li, Qiang Zhou

**Affiliations:** 1 Clinical Laboratory Medicine Department, The Second Affiliated Hospital, Guangzhou Medical University 26468, Guangzhou, China; 2 Department of Pulmonary and Critical Care Medicine, Zhuhai People’s Hospital (The Affiliated Hospital of Beijing Institute of Technology, Zhuhai Clinical Medical College of Jinan University) 198044, Zhuhai, China; 3 Guangdong Provincial Key Laboratory of Tumor Interventional Diagnosis and Treatment, Zhuhai People’s Hospital (The Affiliated Hospital of Beijing Institute of Technology, Zhuhai Clinical Medical College of Jinan University) 198044, Zhuhai, China; Tel Aviv Sourasky Medical Center, Tel Aviv, Israel; Zhejiang Provincial People's Hospital, Hangzhou, Zhejiang, China

**Keywords:** *Klebsiella *spp., *bla*_IMP_, IncN-type plasmid, conjugative transfer region, genetic context

## Abstract

The spread of carbapenem-resistant *Klebsiella* spp. poses a significant public health threat, partly due to the acquisition of the *bla*_IMP_ genes, which encode IMP-type metallo-β-lactamases. These enzymes confer resistance to a broad spectrum of β-lactam antibiotics, including carbapenems, thereby complicating treatment options. This study aims to provide a comprehensive genomic characterization of *bla*_IMP_-harboring plasmids across different species within the genus *Klebsiella*, based on the genomic characteristics of the plasmid pT117-2 of *Klebsiella variicola* strain T117 isolated from clinical settings in China, along with all available *bla*_IMP_-harboring plasmids of *Klebsiella* spp. fromthe GenBank database until 26 April 2025. Among the 123 *bla*_IMP_-harboring plasmids of *Klebsiella* spp., nine variants were identified, with *bla*_IMP-4_ (carried by 69 plasmids) and *bla*_IMP-1_ (carried by 37 plasmids) being the most prevalent. The *bla*_IMP-4_ gene was associated with IncN type (~50 kb, conjugative) and untypeable (~300 kb, non-mobilizable) plasmids in China, whereas in Australia, it was linked to IncC (~200 kb) and IncM2 (~80 kb) type conjugative plasmids. Meanwhile, *bla*_IMP-1_ was found to be associated with IncN (~50 kb), IncM (~80 kb), and IncFII (80 ~200 kb) type conjugative plasmids mainly in Japan. Notably, our results highlight the prevalence of IncN-type conjugative plasmids, including the plasmid pT117-2 identified in this study, as key vehicles for the dissemination of *bla*_IMP_ genes. This study provides critical insights into the genetic mechanisms of *bla*_IMP_-harboring plasmids persistence and spread in *Klebsiella* spp., advancing our understanding of their dissemination.

**IMPORTANCE:**

Carbapenem-resistant *Enterobacterales* (CRE) mediated by metallo-β-lactamases (MBLs) pose a major global public health threat that challenges clinical antimicrobial therapy; based on our study, *bla*_IMP-4_ in China is predominantly associated with IncN plasmids (forming the “IncN-*bla*_IMP-4_-*qnrS1*” axis), while *bla*_IMP-1_ in Japan links to IncN/IncM/IncFII plasmids, with these regional differences highlighting the need for geographically targeted surveillance, and notably, the high-risk ST146 *Klebsiella variicola* carrying *bla*_IMP-4_ on a conjugative IncN plasmid serves as an underrecognized reservoir for resistance genes, extending surveillance beyond common pathogenic *Enterobacterales*; limitations of this study include restricted sample size and geographic scope, and future research should validate these patterns via multi-center studies, explore plasmid evolution mechanisms, and integrate findings into routine surveillance to optimize antibiotic stewardship and infection control, thereby mitigating the global spread of MBL-mediated CRE.

## INTRODUCTION

Carbapenem-resistant *Enterobacterales* (CRE) represent one of the most critical threats to global public health, with the World Health Organization designating them as priority pathogens requiring urgent attention (1, 2). CRE constitutes a large group of bacteria with different mechanisms for drug resistance. Among them, carbapenem-resistant *Klebsiella pneumoniae* accounts for approximately 60%, followed by *Escherichia coli* and *Enterobacter cloacae* (3, 4), but data on carbapenem-resistant *Klebsiella variicola* are limited (5).

Among the major carbapenemase classes, IMP-type metallo-β-lactamases (MBLs) exhibit unique genetic plasticity and transmissibility (6, 7), yet the global dissemination patterns of IMP-MBLs, especially in *Klebsiella* spp., remain poorly understood. First identified in 1991 in *Pseudomonas aeruginosa* isolates from Japan, IMP carbapenemases have since diversified into 96 known variants (8, 9), evolving from regional endemic foci in the Asia-Pacific region to a global concern (5, 10, 11), exacerbating the challenge of treating infections due to their resistance to even novel agents such as cefepime-taniborbactam, which are effective against other MBLs (12, 13).

*bla*_IMP_ dissemination is facilitated by two synergistic processes: (i) horizontal transfer via mobile genetic elements (MGEs), such as conjugative plasmids, and (ii) clonal expansion of high-risk lineages (6, 10, 14, 15). *bla*_IMP_ along with other antimicrobial resistance genes (ARGs) are often located within class 1 integrons carried by broad-host-range plasmids, including IncF, IncC, IncM, IncH, and IncN plasmids in *Enterobacteriaceae* (16). In China, the *bla*_IMP_ genes have been reported in *Klebsiella pneumoniae* belonging to different high-risk sequence types (STs), ST11, ST15, and ST307 (6). Among these, *bla*_IMP-4_ is the most common variant in *bla*_IMP_-producing *Klebsiella* spp., frequently located on IncN plasmids (6). *bla*_IMP-1_ was predominantly identified in Eastern and Southeastern Asia, with Japan being a notable region among the 939 *bla*_IMP-1_ genomes out of 1,155 analyzed (5). In Australia, *bla*_IMP-4_ spreads both clonally and via mobile genetic elements (MGEs), such as broad-host-range plasmids (10). Specific plasmid types have been implicated in regional endemicity, such as IncN-ST7 plasmids, particularly in China (3, 5, 17). These plasmids often harbor insertion sequences (e.g., IS*1*, IS*6100*, IS*26*) and transposons (e.g., Tn*As3*) that facilitate the mobilization of ARGs, including *bla*_IMP-4_ and *qnrS1*, enhancing their spread across bacterial populations (10, 15, 18).

Notably, *K. variicola* is increasingly reported in clinical settings (13, 19). While *bla*_IMP_ epidemiology is well-characterized in *K. pneumoniae* (5, 6), the role of non-pneumoniae species such as *K. variicola* as reservoirs for high-risk IncN plasmids remains under-explored. In China, where *bla*_IMP-4_ is increasingly detected among *Enterobacteriaceae* in hospitals (17, 20), the genomic features of *bla*_IMP-4_-producing *Klebsiella* spp. and the transmission pathways of *bla*_IMP-4_ remain inadequately characterized. Similarly, regional surveillance in Hong Kong and Shanghai has provided localized insights (9, 17, 20, 21). However, integrated genomic analyses of *bla*_IMP_-harboring plasmids across *Klebsiella* species, including emerging hosts such as *K. variicola,* remain scarce, particularly regarding IncN plasmid lineages in China (10, 13, 14).

In this study, we hypothesized that IncN plasmids carrying *bla*_IMP-4_ form a dominant and phylogenetically coherent lineage within *Klebsiella* spp. First, we isolated and characterized a *bla*_IMP-4_-harboring clinical isolate, *K. variicola* strain T117. Second, to contextualize this isolate within the global and Chinese epidemiological landscape, we conducted large-scale comparative genomic and phylogenetic analyses on all 123 available complete plasmid sequences harboring *bla*_IMP_ from the *Klebsiella* spp. in the NCBI database. This dual approach enables us to provide a detailed genomic characterization of a clinically relevant plasmid while simultaneously depicting the broader evolutionary trajectories and transmission patterns of these key resistance determinants. These findings provide an improved understanding of the dissemination characteristics of *bla*_IMP_-harboring plasmids.

## MATERIALS AND METHODS

### Species identification and antimicrobial susceptibility testing

*K. variicola* T117 was collected from a sputum sample of a male patient with severe pneumonia at the Second Affiliated Hospital of Guangzhou Medical University. Species identification was conducted using matrix-assisted laser desorption ionization time-of-flight mass spectrometry (MALDI-TOF/MS) (Bruker Daltonik GmbH, Bremen, Germany), and the carbapenemase gene *bla*_IMP-4_ was detected by PCR (forward primer 5′-GAAGGCGTTTATGTTCATAC-3′ and reverse primer 5′-GTACGTTTCAAGAGTGATGC-3′).

The microbroth dilution method was utilized for antimicrobial susceptibility testing (AST), with *Escherichia coli* ATCC 25922 and *Klebsiella pneumoniae* ATCC BAA-1705 serving as the quality control strains. AST results were interpreted according to the guidelines provided by the Clinical and Laboratory Standards Institute (CLSI 2025) and the European Committee on Antimicrobial Susceptibility Testing (EUCAST 2024) (22).

### Whole genome sequencing and bioinformatic analyses

Whole genome sequencing of T117 strain was performed using paired-end sequencing with Illumina Novaseq (2 × 150 bp paired-end reads), and long sequencing was performed with PacBio Sequel II (23) by Shanghai Biozeron Biotechnology Co., Ltd. (Shanghai, China). PacBio reads were assembled using Unicycler v0.4.8 and polished by Pilon v1.22m with default parameters(3). The assembled genome of *K. variicola* T117 was submitted to the NCBI GenBank database and annotated using the NCBI Prokaryotic Annotation Pipeline (24). Species identification, detection of antimicrobial resistance/virulence genes, MLST typing, and serotype prediction of *Klebsiella* spp. strains were performed using Kleborate software v3.0.8 with default parameters (25). ARGs were identified using ResFinder v4.7.2 (26). Plasmid replicon types were determined using PlasmidFinder v2.1 (27). Integrons and insertion sequence elements were disclosed using ISfinder (28), respectively. Comparative analyses of different plasmid genome sequences were performed using the BLAST Ring Image Generator (BRIG v0.95) (29).

### Plasmid characterization and conjugation experiment

The location of *bla*_IMP-4_ on the plasmid pT117-2 was determined through ResFinder v4.7.2 (30). To investigate the transfer ability of the plasmid, *E. coli* J53 was employed as the recipient strain in conjugation experiments. Transconjugants were selected using Mueller-Hinton agar (OXOID, Hampshire, United Kingdom) supplemented with 200 mg/L NaN_3_ and 2 mg/L meropenem. Their identification as *E. coli* and the presence of the *bla*_IMP-4_ genes were confirmed through PCR and further validated using MALDI-TOF/MS.

### Identification of the *bla*_IMP_-harboring plasmids of *Klebsiella* genus

We performed MegaBLAST (31) analysis by querying the *bla*_IMP-4_ gene sequence against the GenBank nonredundant (nr) genus database on 26 April 2025. The primary objective of this analysis was to identify plasmids carrying the *bla*_IMP_ gene, with application of filtering thresholds set at 95.0% sequence coverage and >83.8% nucleotide identity for *bla*_IMP_-harboring plasmids. We selected the circular plasmids with complete sequence that were taxonomically classified under the genus *Klebsiella* for subsequent comparative genomic analysis. The variants of *bla*_IMP_ genes were identified using ResFinder software (26) and the beta-lactamase database (32).

### Geographic location and origin of *bla*_IMP_-harboring plasmids of *Klebsiella* spp.

Geographic location and origin information for *bla*_IMP_-harboring plasmids within the *Klebsiella* spp. were extracted in batches from the “source” section of corresponding genomic files in GenBank format, using the key words “geo_loc_name,” “isolation_source,” and “host”. For plasmids lacking relevant records on the geographic location and origin, we searched for the corresponding information in their published papers according to the PubMed ID (PMID) numbers included in the GenBank files. To ensure data accuracy, only information obtained through two methods was included in our analyses.

### Bioinformatic analyses of the *bla*_IMP_-harboring plasmids of *Klebsiella* spp.

For phylogenetic cladogram of the *bla*_IMP_-harboring plasmids from *Klebsiella* spp., a binary matrix of orthologous gene family presence/absence was constructed with OrthoFinder (33), followed by hierarchical clustering via PAST3 (34) and visualization of the resulting phylogenetic cladogram using iTOL (35). Plasmid replicon typing was performed with PlasmidFinder 2.1 (27) against the *Enterobacteriales* database (updated 29 November 2021) at parameters of 95% minimum identity and 60% minimum coverage, using FASTA-formatted plasmid genomic files for batch analysis. Conjugative transfer regions were analyzed by processing GenBank-formatted plasmid files in batches with the standalone version of oriTfinder2 (36) to identify the presence/absence of *oriT*s, relaxase genes, T4CP genes, and T4SS gene cluster. Bacterial insertion sequences within the plasmids were explored using ISfinder (28), and comparisons of the genetic contexts surrounding *bla*_IMP_ genes were conducted using BLAST Ring Image Generator (29).

## RESULTS

### Isolation and genomic characteristics of *K. variicola* T117

*K. variicola* strain T117 was isolated from a sputum sample collected on 18 January 2024, from a 77-year-old male patient diagnosed with severe pneumonia. Initial species identification via MALDI-TOF/MS was confirmed as *K. variicola* using whole-genome sequencing combined with the Kleborate bioinformatics tool.

Whole genome sequencing revealed the strain harbors a circular chromosome (5,564,478 bp, GenBank accession: CP193999) and two plasmids: pT117-1 (146,290 bp GenBank accession: CP194000) and pT117-2 (52,439 bp GenBank accession: CP194001). The total genome encodes 5,608 genes, including 5,356 protein-coding CDSs and 125 RNA genes (85 tRNAs, 25 complete rRNAs [9 5S, 8 16S, 8 23S], and 15 ncRNAs), with the majority of protein-coding genes localized to the chromosome. Whole genome sequence analysis via Kleborate software revealed that strain T117 belongs to *K. variicola*, with K_locus KL103 and sequence type ST146-4LV. PCR amplification with *bla*_IMP_-specific primers yielded a 587 bp fragment, and Sanger sequencing confirmed the presence of the carbapenemase-encoding gene *bla*_IMP-4_. Six ARGs were identified in the strain: β-lactamase genes (*bla*_LEN-22_, *bla*_IMP-4_), fosfomycin resistance gene (*fosA*), quinolone resistance gene (*qnrS1*), and efflux pump genes (*oqxA*, *oqxB*) (Table 1). Among these, *bla*_IMP-4_ and *qnrS1* were mapped to pT117-2, identifying it as a multidrug-resistant (MDR) plasmid (Table 2).

**TABLE 1:** ARGs of *K. variicola* T117^*a*^

Genome	Drug class	Genes	Location
Chromosome	Beta-lactam	*bla*_LEN22_	2,837,564...2,838,424
	Fosfomycin	*fosA*	4,861,584...4,862,003
	Efflux pump-associated gene	*oqxB*	1,170,641...1,173,793
	Efflux pump-associated gene	*oqxA*	1,173,817...1,174,992
pT117-1	None	None	–
pT117-2	Quinolone	*qnrS1*	3290...3946
	Beta-lactam	*bla*_IMP-4_	32,362...33,102

^*a*^ “None” represents absence of ARGs; “–” represent no data at the indicated position.

**TABLE 2:** Characteristics of pT117-2 harboring *bla*_IMP-4_^*a*^

Type	Locus tag/Gene name	Location
*oriT* region	–	13,508...14,134 (+)
Relaxase	orf13	8,415...11,651 (−)
T4CP	orf14	11,651...13,180 (−)
Auxiliary protein	Protein 15	13,182...13,316 (−)
T4SS gene cluster	orf57: orf59: orf60: orf61: orf62: orf64: orf65: orf66: orf67	42,115..,51,403
ARGs	*qnrS1*; *bla*_IMP-4_	3,623...3,946 (+); 32,362...33,102 (+)

^*a*^ “–” indicate unidentified or unannotated *oriT*s.

### Genomic characterization and novelty of plasmid pT117-2

Plasmid pT117-2 (CP194001) is 52,439 bp in length, encodes 65 protein-coding genes, and has a G + C content of 51.07%. The plasmid pT117-2 was assigned to the IncN type by PlasmidFinder analyses.

NCBI BLAST alignment revealed no complete sequence matches to known plasmids, confirming the novelty of pT117-2. However, it shared high sequence identity with pIMP-SZ1502 (KU051707), an *E. coli*-derived plasmid isolated in Shenzhen, China, suggesting potential evolutionary relatedness. Genetic context analysis showed *bla*_IMP-4_ was flanked by class one integrase (*IntI1*) and *ltrA* (forming the *IntI1-bla*_IMP-4_*-ltrA* cassette), a structure typical of integron-mediated ARG capture.

### Antimicrobial susceptibility profiles: T117 vs transconjugant T117-J53

Antimicrobial susceptibility testing and conjugation experiments clarified the role of pT117-2 in resistance phenotypes (Table 3).

**TABLE 3:** MIC values of antimicrobials for *K. variicola* T117, transconjugant T117-J53, and recipient strain *E. coli* J53^*a*^^,^^*b*^

Antibiotic	MIC values (mg/L)		
Antimicrobials	T117	T117-J53	J53
Ceftazidime	≥64/R	≥64/R	≤0.12/S
Cefepime	8/SDD	4/SDD	≤0.12/S
Aztreonam	≤1/S	≤1/S	≤1/S
**Imipenem**	**≥8/R**	**≥16/R**	**≤0.25/S**
**Meropenem**	**≥16/R**	**≥16/R**	**≤0.25/S**
Amikacin	≤2/S	≤2/S	≤2/S
Tobramycin	≤1/S	≤1/S	≤1/S
**Ciprofloxacin**	**≥4/R**	**1/R**	**≤0.25/S**
**Levofloxacin**	**≥8/R**	**1/I**	**≤0.12/S**
Doxycycline	≥16/R	2/S	2/S
Minocycline	≥16/R	2/S	2/S
Tigecycline	≥8/R	≤0.5/S	≤0.5/S
Colistin	≤0.5/S	≤0.5/S	≤0.5/S
Ticarcillin/clavulanic acid	≥128/R	≥128/R	≤8/S
Piperacillin/tazobactam	≥128/R	≥128/R	1/S
Cefoperazone/sulbactam	≥64/R	≥64/R	≤8/S
Trimethoprim/sulfamethoxazole	≤20/S	≤20/S	≤20/S

^*a*^ S, susceptible; I, intermediate; R, resistant; SDD, susceptible dose-dependent. SDD indicates that therapeutic efficacy relies on optimized dosage regimens to achieve adequate drug exposure at the infection site.

^*b*^ Bold values represent MICs of carbapenem antibiotics, which are emphasized to facilitate comparison of susceptibility profiles before and after conjugative transfer.

**Fig 1:**
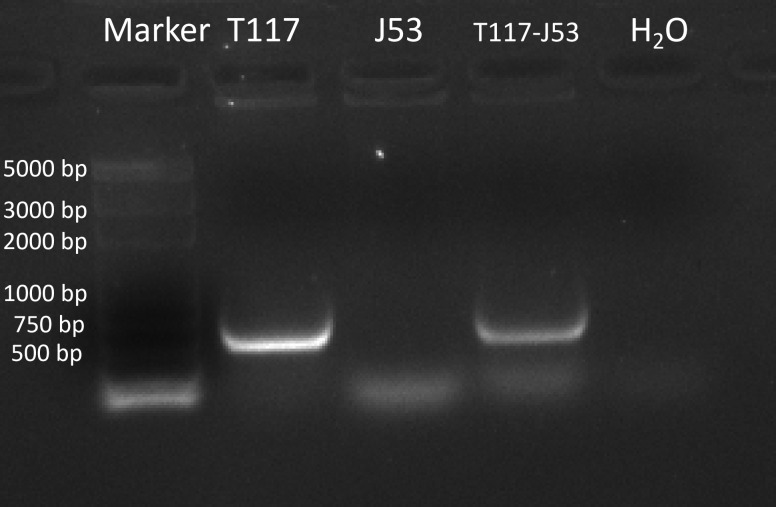
PCR identification of T117-J53 (the conjugative transconjugant of *K. variicola*-derived *bla*_IMP_-carrying plasmid pT117), *K. variicola* strain T117, and *E. coli* J53 were used as positive and negative controls, respectively. Agarose gel electrophoresis was performed to verify the target fragment after PCR amplification. Lane M contains a DNA ladder with known fragment sizes, serving as a reference for molecular weight determination. The specific bands in the experimental lanes (Lanes 1–4) exhibited consistent migration positions of PCR reactions for templates of T117, *E. coli* J53, the T117-J53 transconjugant, and H_2_O, which matched the expected size of the target sequence.

Both T117 and T117-J53 exhibited resistance to broad-spectrum β-lactams (ceftriaxone, cefotaxime, ceftazidime, cefepime), β-lactam/β-lactamase-inhibitor combinations (piperacillin–tazobactam, amoxicillin–clavulanic acid, ticarcillin–clavulanate) with MICs ≥ 128 µg/mL, sulfonamides (trimethoprim/sulfamethoxazole), and gentamicin (Table 3). Both strains remained susceptible to amikacin and colistin (Table 3). High-level carbapenem resistance in T117-J53 (imipenem MIC ≥ 8 µg/mL, meropenem MIC ≥ 16 µg/mL) confirmed *bla*_IMP-4_ mediates carbapenem resistance refractory to classical inhibitors, consistent with prior observations in *K. variicola* strain T117. PCR and electrophoresis further validated successful transfer of functional *bla*_IMP-4_ (Fig. 1).

Notable phenotypic discrepancies between T117 and T117-J53 indicated the presence of non-plasmid-borne resistance determinants: while T117 exhibited resistance to tigecycline and fosfomycin, T117-J53—having only inherited the conjugative plasmid pT117-2—was susceptible to all tested tetracyclines (doxycycline, minocycline, tigecycline) as well as fosfomycin. These findings confirm that the genetic determinants underlying tigecycline and fosfomycin resistance are not localized on the conjugative, multidrug-resistant pT117-2; instead, they likely reside on the bacterial chromosome or the non-conjugative plasmid pT117-1, highlighting the modular distribution of ARGs in this clinical *K. variicola* isolate T117.

### Genomic diversity of the *bla*_IMP_-harboring plasmids in *Klebsiella* spp.

A total of 123 plasmids carrying *bla*_IMP_ genes available in the GenBank database, including the plasmid pT117-2 identified in this study, were included in this study.

**Fig 2:**
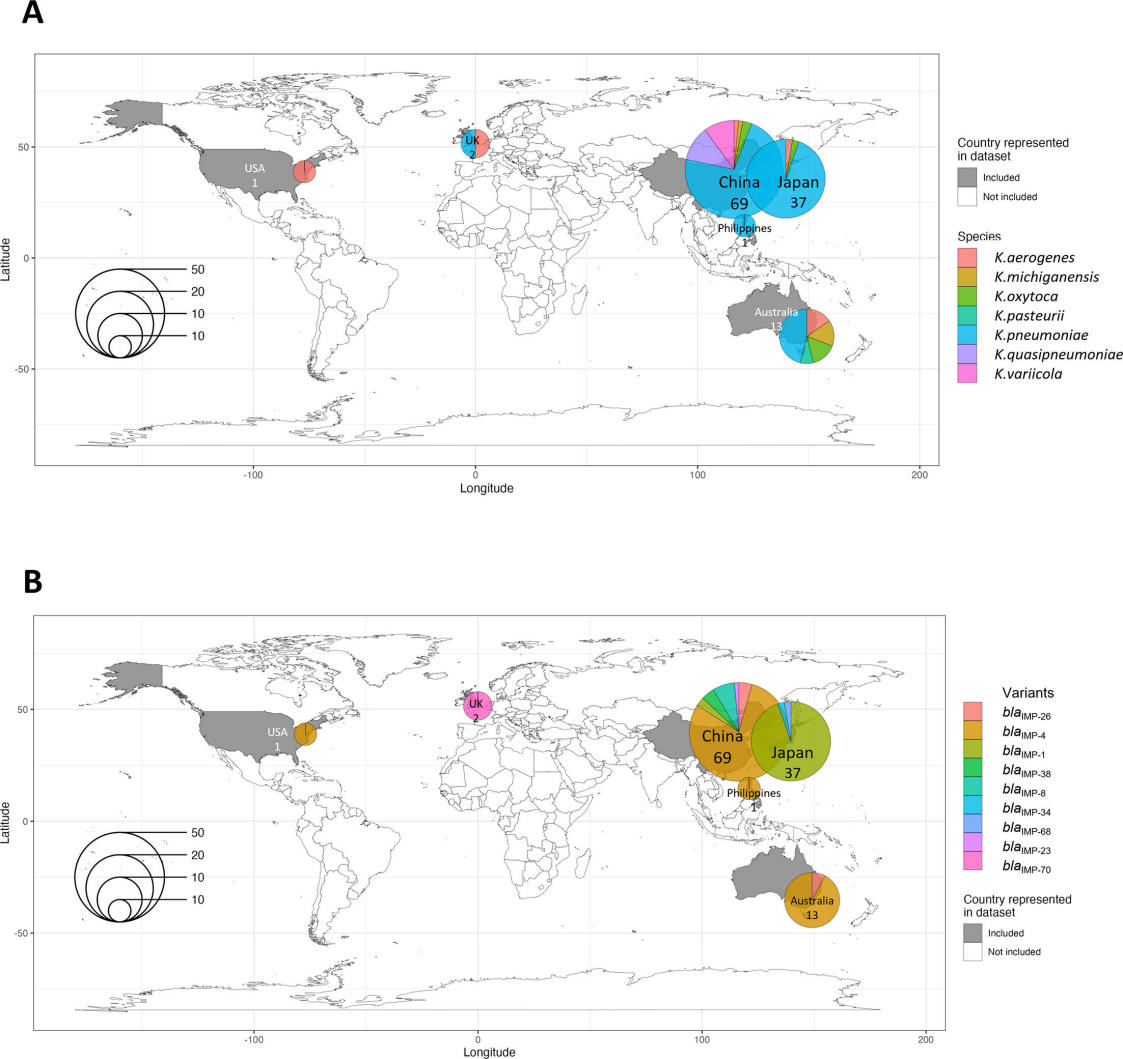
Geographic distribution of *bla*_IMP_ variants in *Klebsiella* spp. (**A**) Epidemiology of *bla*_IMP_-harboring *Klebsiella* spp., showing regional endemicity in Asia and other geographic regions. (**B**) Global distribution of *bla*_IMP_ variants in *bla*_IMP_-harboring plasmids, with panels showing regional endemicity concentrated within East Asia. The color shading represents the hosts of *Klebsiella* spp. (**A**) or variants of *bla*_IMP_-harboring plasmids (B). The diameter of pie charts reflects the proportion of representative strains per region.

Among the 123 *bla*_IMP_-harboring plasmids, China had the highest number of isolates (65 plasmids), followed by Japan (37 plasmids) and Australia (13 plasmids) (Fig. 2A). Other regions, such as Taiwan (3) and Hong Kong (1) of China, the UK (2), the USA (1), and the Philippines (1) had relatively fewer strains (Fig. 2A).

The 123 *bla*_IMP_-harboring plasmids were found in seven different host species (Fig. 2A). *K. pneumoniae* was the most common host species, with 93 strains (75.6%), of which 47 were from China and 35 from Japan. The other species had relatively few *bla*_IMP_-harboring strains: *K. quasipneumoniae* (8, 6.5%), *K. variicola* (7, 5.7%), *K. aerogenes* (6, 4.9%), *K. oxytoca* (5, 4.1%), *K. michiganensis* (3, 2.4%), and *K. pasteurii* (1, 0.8%).

**Fig 3:**
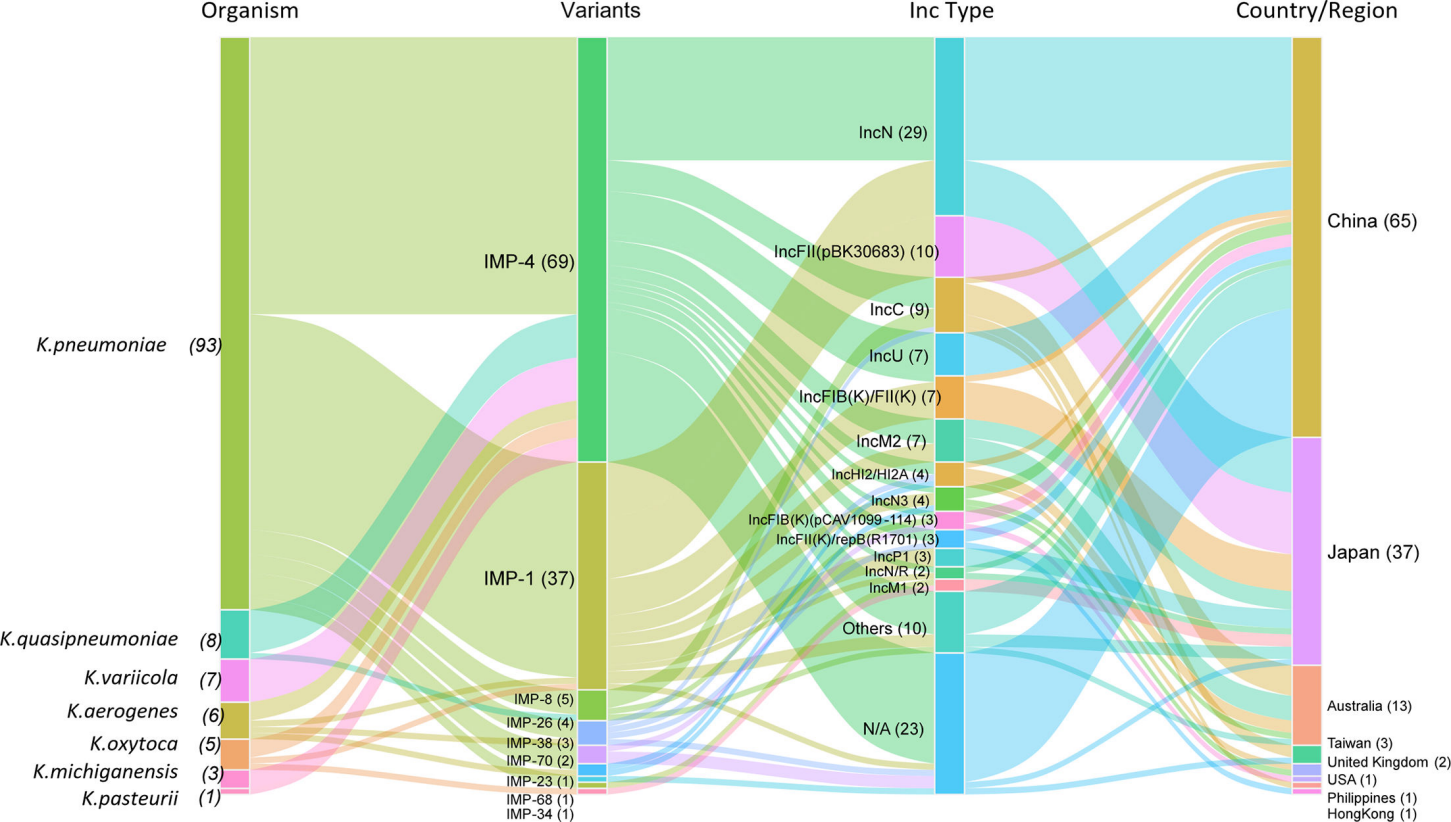
Sankey diagram showing the correspondence between *bla*_IMP_ variants, Inc types, organism hosts, and isolated geographic distribution. Connecting ribbon width is proportional to isolate count. *K. pneumoniae* was the dominant species, with *bla*_IMP-4_ and *bla*_IMP-1_ as the major gene variants. IncN, IncF, and IncC were the primary plasmid Inc types, and isolates were predominantly distributed in China, Japan, and Australia. This diagram visualizes key epidemiological correlations for *bla*_IMP_ resistance gene dissemination.

Nine *bla*_IMP_ variants were detected in the 123 *bla*_IMP_-harboring plasmids, with *bla*_IMP-4_ (69 plasmids) and *bla*_IMP-1_ (37 plasmids) being the most prevalent (Fig. 2 and 3). *bla*_IMP-4_ demonstrated a strong tropism for *K. pneumoniae*, being identified in 46 out of 69 plasmids (66.7%). In contrast, *bla*_IMP-4_ was sparsely distributed in other species: *K. quasipneumoniae* (7 isolates, 10.1%), *K. variicola* (7, 10.1%), *K. aerogenes* (3, 4.3%), *K. oxytoca* (3, 4.3%), *K. michiganensis* (2, 2.9%), and *K. pasteurii* (1, 1.4%). *bla*_IMP-1_ also showed a strong preference for *K. pneumoniae*, being identified in 36 out of 37 plasmids (97.3%). Other ARGs such as *bla*_IMP-8_ and *bla*_IMP-26_ and species, such as *K. aerogenes* and *K. variicola,* were sparsely distributed across the sampled regions (Fig. 2B).

Analysis of plasmid types revealed that IncN was the predominant vector for *bla*_IMP_, identified in 33 plasmids. Other plasmid types included IncC (9 plasmids), IncF (13 plasmids), IncM (9 plasmids), IncU (7 plasmids), IncH (3 plasmids), and IncP (3 plasmids). It is evident that the association of *K. pneumoniae* with *bla*_IMP-4_ and IncN plasmids is predominant in China (Fig. 3). However, the *bla*_IMP-4_-carrying IncN-type plasmid pT117 from *K. variicola* in China highlights the potential for *bla*_IMP-4_ to spread beyond the primary reservoir species.

### Phylogenetic cladogram of the *bla*_IMP_-harboring plasmids in *Klebsiella* spp.

For the 123 *bla*_IMP_-harboring plasmids in *Klebsiella* spp., 122 intact plasmids were finally analyzed and classified into eight major clades (Clade I–VIII) based on phylogenetic cladogram, *bla*_IMP_ variants, host species, replicon type, origin of replication, conjugation elements, and plasmid size. The characteristics of each lineage are as follows:

#### Clade I: conjugative IncN-type *bla*_IMP_-carrying plasmids

**Fig 4:**
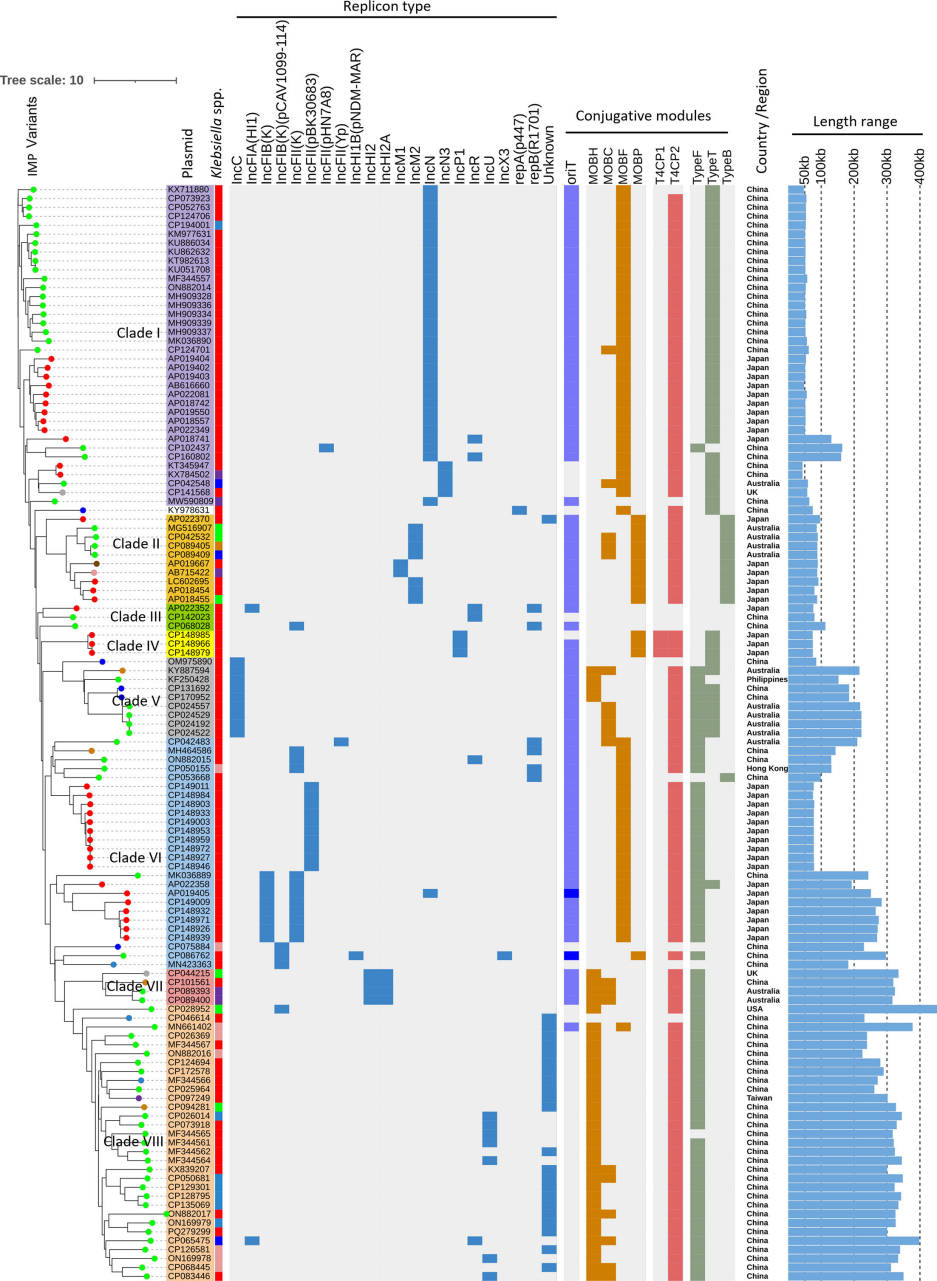
Details of variants of *bla*_IMP_ genes, replicon types, conjugative transfer regions, isolated regions and length distribution of the 123 *bla*_IMP_-harboring plasmids of *Klebsiella* spp. The seven categories of information present in this figure include the phylogenetic cladogram, variants of *bla*_IMP_, the isolated organism hosts, replicon types, conjugative transfer regions (*oriT*, relaxase, T4CP, and T4SS), host isolated regions, and length distribution of the 123 *bla*_IMP_-harboring plasmids of *Klebsiella* spp.

**Fig 5:**
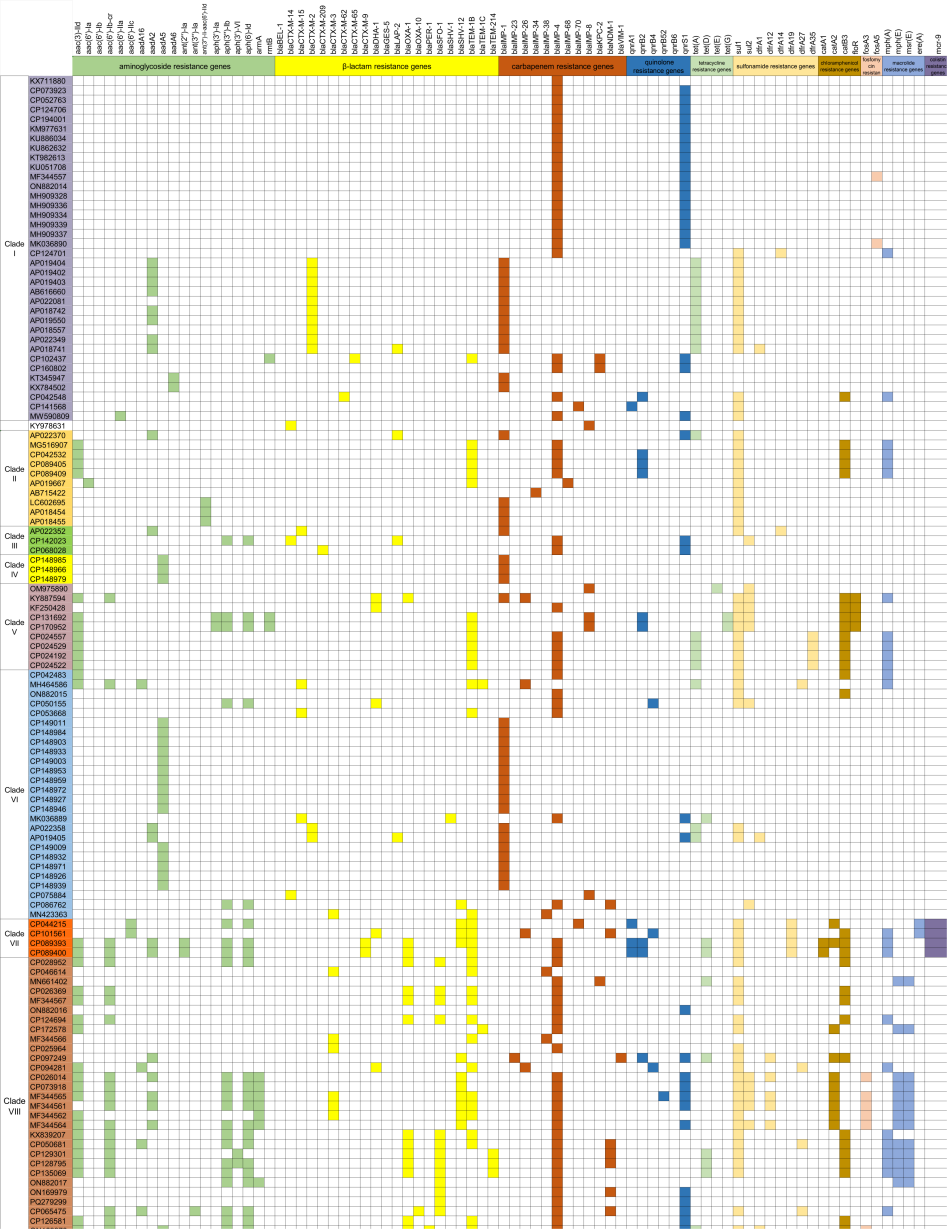
Heatmap showing the distributions of ARGs in the *bla*_IMP_-harboring plasmids of *Klebsiella* spp. The *x*-axis represents resistance gene families (e.g., aminoglycoside, β-lactam, carbapenem, quinolone ARGs), and the *y*-axis denotes plasmid identifiers and clades. The squares are colored according to the drug resistance categories, represent the presence of the trait examined. Color indicates the relative resistance gene, with distinct enrichment patterns observed for carbapenem and β-lactam ARGs in specific plasmids, reflecting clade specificity in resistance gene profiles.

**Fig 6:**
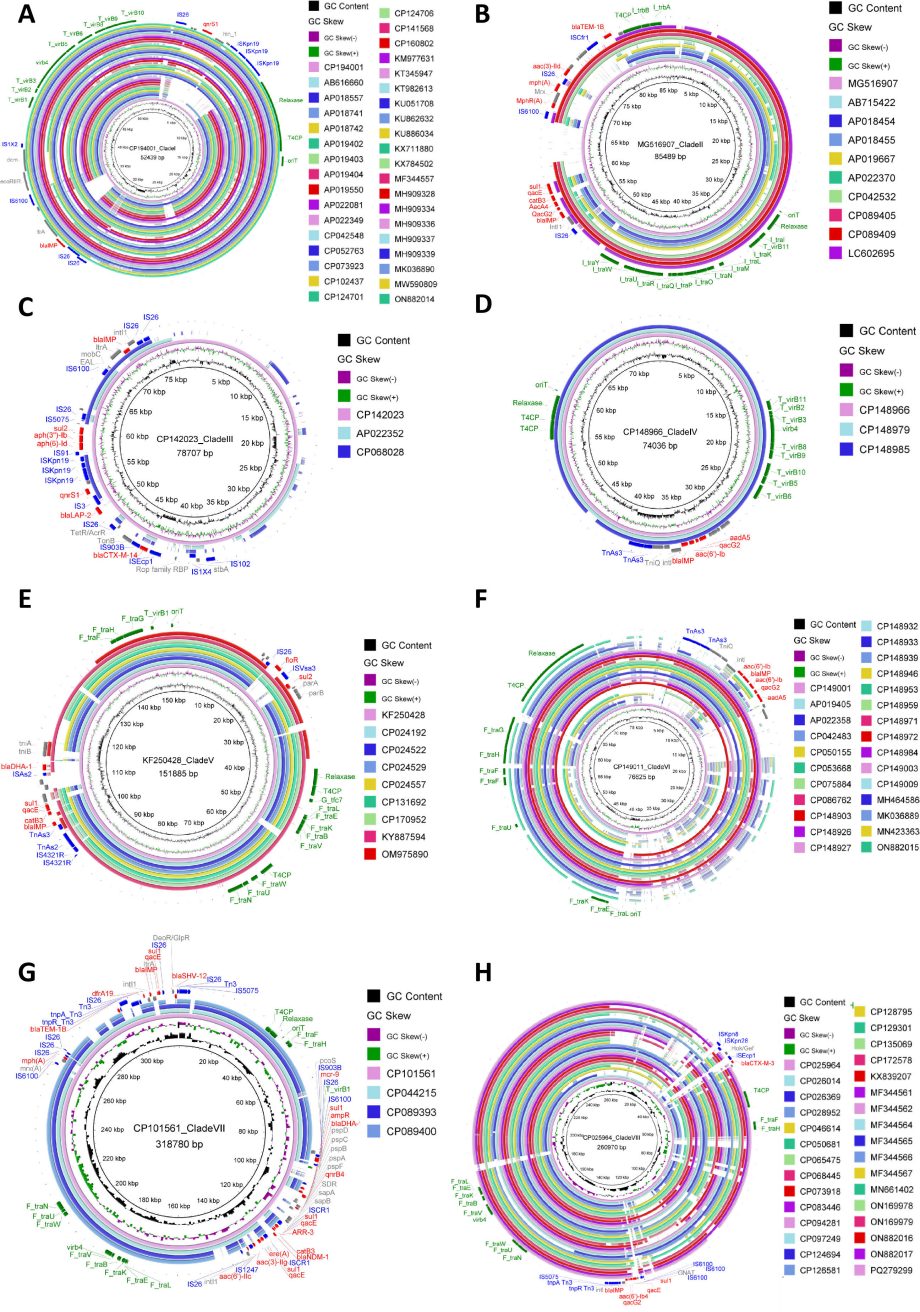
Comparative analysis of the eight clades of the *bla*_IMP_-harboring plasmids generated by BRIG. The ORFs of different functions are represented in various colors on the map. Backbone regions of the plasmid and some of the important genes of the plasmids are plotted on the map. (**A**) Clade I: IncN-type plasmids. (**B**) Clade II: IncM-type plasmids. (**C**) Clade III: IncR-type plasmids. (**D**) Clade IV: IncP-type plasmids. (**E**) Clade V: IncC-type plasmids. (**F**) Clade VI: IncF-type plasmids. (G) Clade VII: IncH-type plasmids. (H) Clade VIII: IncU-type and untypeable plasmids. ARGs, transposase, genes encoding for conjugative transfer elements and other genes surround the ARGs are shown in red, blue, green, and gray arrows, respectively. The purple shades denote transposons. Arrows indicate the translation orientation of the coding genes.

Among 123 *bla*_IMP_-carrying plasmids, 36 were classified into Clade I, primarily consisting of plasmids harboring *bla*_IMP-4_ and *bla*_IMP-1_ from *Klebsiella pneumoniae* (Fig. 4), accounting for 29.3% of all *Klebsiella* plasmids carrying *bla*_IMP_. IncN-type plasmids carrying *bla*_IMP-4_ typically harbor the quinolone resistance gene *qnrS1*, while plasmids carrying *bla*_IMP-1_ are often associated with five additional ARGs (Fig. 5). Notably, one plasmid carrying *bla*_IMP-70_ was also classified into Clade I (Fig. 4). Among the *bla*_IMP_-carrying IncN plasmids, the most common replicon type is IncN, followed by four individual IncN3 plasmids (Fig. 4). The plasmids are generally around 50 kb in size, ranging from 42.46 to 163.39 kb (Fig. 4). In the conjugative transfer region, most Clade I plasmids were found to harbor a single *oriT*, along with a MOB_F_-family relaxase gene, T4CP2 gene, and T-type T4SS gene cluster (Fig. 4 and 6A), suggesting these are conjugative plasmids. Members of Clade I are distributed worldwide, with a majority concentrated in China (24 plasmids) and Japan (10 plasmids), with isolated plasmids from Australia and the United Kingdom (one plasmid each) (Fig. 4). We also analyzed the genetic environment of the 36 *bla*_IMP_-carrying IncN plasmids in genus *Klebsiella*, and found that the *bla*_IMP_ gene is often surrounded by IS*6100* and IS*26* elements, forming a highly conserved “IS6100-*mobC-ltrA-bla*_IMP-4_-*∆IntI1*-IS*26*” cassette (Fig. 6A).

#### Clade II: conjugative IncM-type *bla*_IMP_-carrying plasmids

Clade II represents IncM-type plasmids, including seven IncM2-type, two IncM1-type, and one untypeable type, as shown in Fig. 4. These plasmids exhibit a distinct geographical distribution, primarily originating from Japan (6 out of 10) and Australia (4 out of 10) (Fig. 4). Unlike Clade I, the *bla*_IMP_ variants in this clade are more diverse, including *bla*_IMP-1_, *bla*_IMP-4_, *bla*_IMP-68_, and *bla*_IMP-34_ (Fig. 4). In addition to *bla*_IMP_, most of these plasmids also harbor ARGs for aminoglycosides, β-lactams, and sulfonamides (Fig. 5). The plasmids were isolated from *K. pneumoniae* (4), *K. aerogenes* (3), *K. michiganensis* (1), *K. pasteurii* (1), and *K. oxytoca* (1) (Fig. 4). The plasmid sizes ranged from 79.00 to 96.04 kb (Fig. 4). All plasmids in this group contain a single *oriT*, along with MOB_p_-family relaxase gene, T4CP2 gene and peculiar B-type T4SS (Fig. 4 and 6B), suggesting that they are conjugative plasmids. We also analyzed the genetic environment of the *bla*_IMP_ gene in these plasmids. The *bla*_IMP_ gene is surrounded by ARGs, such as *sul1* and *qacE*, as well as integron 1 and IS*26* elements, which is therefore enclosed in the In*809* structure (*intl1-bla*_IMP-4_-*qacG2-aacA4-catB3-qacE1-sul1*) (Fig. 4 and 6B).

#### Clade III: non-mobilizable *bla*_IMP_-carrying plasmids with multi-replicon

Three plasmids were classified into Clade III, with one being of the IncFIA(HI1)/R/repB(R1701) type, the other is IncR type, and the third of the IncFII(K)/repB(R1701) type. In addition to carrying *bla*_IMP_, the three plasmids also harbor the *bla*_CTX-M_ gene (Fig. 4 and 6C). One plasmid carries *bla*_IMP-1_, while the other two carries *bla*_IMP-4_ (Fig. 4). All three plasmids were isolated from *K. pneumoniae* (Fig. 4). The plasmid sizes were 75.53, 78.71, and 112.21 kb, respectively (Fig. 4). None of these plasmids contains gene encoding relaxase, T4CP, or T4SS, indicating that they are not conjugative plasmids (Fig. 4 and 6C). Members of Clade III are mainly distributed in Japan (one plasmid) and China (two plasmids) (Fig. 4).

#### Clade IV: IncP-type *bla*_IMP-1_-harboring plasmids

Three plasmids classified as IncP1-type belong to Clade IV, all isolated from *K. pneumoniae*, carrying *bla*_IMP-1_ and *aadA5* (Fig. 4 and 6D). The plasmid sizes are mostly around 74 kb (Fig. 4). In the conjugative transfer region, plasmids from Clade IV were found to harbor a single *oriT*, MOB_P_-family relaxase gene, encoding both T4CP1 and T4CP2 genes and T-type T4SS gene cluster (Fig. 4 and 6D), suggesting these are conjugative plasmids. All members of Clade IV were isolated from Japan. We also analyzed the genetic environment of the *bla*_IMP_ gene in the 3 IncP1-type plasmids from *Klebsiella* spp. The genetic environment of *bla*_IMP_ in this clade is characterized by the arrangement “Tn*As3-TniQ-int-bla*_IMP-1_-*aac(6')-Ib-qacG2-aadA5*” (Fig. 6D).

#### Clade V: conjugative IncC-type *bla*_IMP_-carrying megaplasmids in *K. pneumoniae*

Nine plasmids were classified into Clade V, all isolated from *K. pneumoniae*. Five plasmids carried *bla*_IMP-4_, three carried *bla*_IMP-8_, and one carried *bla*_IMP-26_ (Fig. 4). Most of these plasmids also harbor *aac(3)-IId* and *bla*_TEM-1B_ (Fig. 6E). Among the IncC-type plasmids carrying *bla*_IMP_, four were isolated from Australia, two from Taiwan, China, one from the Philippines, and one from mainland China (Fig. 6E). The plasmid sizes ranged from 84.44 to 221.61 kb (Fig. 4). In the conjugative transfer region, most Clade V plasmids were found to harbor a single *oriT*, a relaxase gene belonging to MOB_C_ or MOB_F_ family, T4CP2 gene and both T-type and F-type T4SS gene clusters (Fig. 6E), suggesting these are conjugative plasmids. We also analyzed the genetic environment of the *bla*_IMP_ gene in the nine IncC-type plasmids from *Klebsiella* spp. The *bla*_IMP_ gene is often surrounded by ARGs such as *sul1*, *qacE*, and *catB3*, as well as the insertion elements Tn*As3* and IS*4321R* (Fig. 6E).

#### Clade VI: conjugative IncF-type *bla*_IMP_-harboring plasmids

Twenty-six plasmids were classified as IncF-type, belonging to Clade VI, almost all of which were isolated from *K. pneumoniae*. Seventeen plasmids carried *bla*_IMP-1_, six carried *bla*_IMP-4_, one carried *bla*_IMP-8_, one carried *bla*_IMP-26_, and one carried *bla*_IMP-38_ (Fig. 4). In addition to *bla*_IMP_, most plasmids also harbor *aadA5*. The plasmid sizes ranged from 74.63 to 296.15 kb (Fig. 4). In the conjugative transfer region, plasmids from Clade VI were found to harbor a single *oriT*, primarily encoding MOB_F_-family relaxase gene, T4CP2 gene and F-type T4SS gene cluster (Fig. 4 and 6F), suggesting that they are conjugative plasmids. Seventeen plasmids from Clade VI were isolated from Japan, seven from China, one from Hong Kong, and one from Australia. We also analyzed the genetic environment of the *bla*_IMP_ gene in the 26 IncF-type plasmids from *Klebsiella* spp. The genetic environment of the *bla*_IMP_ gene in most plasmids was found to be “Tn*As3-TniQ-int-bla*_IMP-1_-*aac(6')-Ib-qacG2-aadA5*” (Fig. 6F), which is similar to the IncP plasmids.

#### Clade VII: conjugative IncH-type *bla*_IMP_-carrying megaplasmids

Four plasmids were classified as IncH-type, belonging to Clade VII. Two were isolated from *K. oxytoca*, one from *K. aerogenes*, and one from *K. pneumoniae*. Two plasmids carried *bla*_IMP-4_, one carried *bla*_IMP-26_, and one carried *bla*_IMP-70_ (Fig. 4). The plasmid sizes ranged from 316.12 to 333.88 kb (Fig. 4). These large plasmids also harbor a variety of other ARGs (Fig. 4). In the conjugative transfer region, plasmids from Clade VII were found to harbor a single *oriT*, primarily encoding relaxase genes both MOB_H_ and MOB_C_-family, T4CP2 gene, and a F-type T4SS gene cluster (Fig. 4 and 6G), suggesting they are also conjugative plasmids. Members of Clade VII were isolated from the United Kingdom of Great Britain and Northern Ireland (UK) (one plasmids), China (one plasmid), and Australia (two plasmid). We also analyzed the genetic environment of the *bla*_IMP_ gene in the four IncH-type plasmids from *Klebsiella* spp. The genetic environment of *bla*_IMP_ in these plasmids is often associated with integron *Intl1*, as well as ARGs *qacE*, *sul1*, and *bla*_TEM-1B_. The plasmids also contain additional resistance gene clusters (Fig. 6G).

#### Clade VIII: conjugative IncU-type and untypeable *bla*_IMP_-harboring megaplasmids

A total of 31 plasmids classified as IncU and untypeable-type belong to Clade VIII. Sixteen plasmids were isolated from *K. pneumoniae*, six from *K. quasipneumoniae*, six from *K. variicola*, two from *K. aerogenes*, and one from *K. michiganensis*. Twenty-seven plasmids carried *bla*_IMP-4_, two carried *bla*_IMP-38_, one carried *bla*_IMP-23_, and one carried *bla*_IMP-26_ (Fig. 4). These plasmids are relatively large, with sizes ranging from 224.75 to 397.45 kb (Fig. 4), and commonly harbor other types of ARGs (Fig. 5). In the conjugative transfer region, plasmids from Clade VIII primarily encoding relaxase genes belong to MOB_H_-family with occasional inclusion of MOB_C_-family, T4CP2 gene and F-type T4SS gene cluster (Fig. 4 and 6H), suggesting that they are conjugative plasmids. The plasmid found to harbor no *oriT*. All members of Clade VIII were isolated from China. We also analyzed the genetic environment of the *bla*_IMP_ gene in these plasmids from *Klebsiella* spp. The genetic environment of the *bla*_IMP_ gene is often associated with ARGs, such as *qacE* and *sul1*, as well as insertion sequences IS*6100* and IS*5075*, and transposons Tn*As2* and Tn*As3*. Additionally, the plasmids carry the *bla*_CTX-M-3_ resistance gene (Fig. 6H).

## DISCUSSION

The ongoing epidemic of carbapenemase-producing *Klebsiella* spp. has emerged as a significant threat to global human health (1, 2). However, due to the fact that KPC, NDM enzymes, and OXA-48-like carbapenemases are the predominant types in CRKP strains in China and have been extensively studied (1, 2), research on the prevalence and genomic characterization of IMP-producing *Klebsiella* spp. isolates is relatively limited nationwide. Since its first identification in Wuhan in 2008, the IMP-type MBL has been associated with sporadic infections and outbreaks of IMP-producing *Klebsiella* spp. strains nationwide, notably with IMP-producing ST307 *K. pneumoniae* causing infections (6). In this study, we identified the presence of the *bla*_IMP-4_ gene on the IncN-type plasmid in *K. variicola* T117. Our analysis of 123 *bla*_IMP_-harboring plasmids revealed a diverse genetic background for *bla*_IMP_ genes, with IncN-type plasmids emerging as the primary vehicle for the dissemination of *bla*_IMP-4_ in China.

*K. variicola*, often found in plants and soil, bridges environmental and clinical settings (37, 38). The ST146 type of *K. variicola*, notable for its prevalence and virulence, was identified in a 2021 Sweden study as the most prevalent sequence type (12.0%) among isolates from patients with suspected community-onset sepsis (39). On April 22, 2025, an ST146 *K. variicola* isolate with β-lactam resistance was found in a diarrhea patient in Kunming, Yunnan (40). The identification of *K. variicola* T117 as ST146-4LV highlights the complexity of its epidemiological profile and underscores the need for global surveillance to track ST146’s spread and assess its public health impact. The association of *bla*_IMP_-carrying IncN plasmids with ST146 *K. variicola*, highlighted by our study’s identification of *K. variicola* T117 with the IncN plasmid pT117-2, suggests a spillover event and warrants increased vigilance to control IMP-mediated carbapenem resistance.

The spread of carbapenemase-encoding genes like *bla*_IMP_, often mediated by plasmids, significantly contributes to the emergence and spread of CRE (21, 41). The IncN-type plasmid lineage, known for its stability and broad-host-range, has successfully integrated into bacterial populations in East Asia (21). Typically around 50 kb, IncN plasmids are key vectors for spreading ARGs due to their broad host range and conjugative ability (5, 17, 42). Our conjugation experiments confirmed that the IncN-plasmid pT117-2 can be transferred from *K. variicola* to *E. coli* J53 through conjugation, mediating the horizontal transfer of ARGs. The geographic origin of T117, isolated in Guangzhou, underscores the concentration of IncN-type plasmids in China. Thus, pT117-2/T117exemplifies the dominant epidemiological trend revealed by our large-scale data analysis: the IncN/*bla*_IMP-4_/*qnrS1*/China axis. A NCBI BLAST search revealed that pT117-2 exhibited high sequence similarity to pIMP-SZ1502 from an *E. coli* isolate in Shenzhen, suggesting potential plasmid horizontal transfer among different genera within the Pearl River Delta region.

In addition, the association of *bla*_IMP_ genes with specific host bacterial species also affects their transferability. For example, *bla*_IMP-1_, *bla*_IMP-4_, and *bla*_IMP-6_ are predominantly associated with *Enterobacterales* (such as *K. pneumoniae* and *E. coli*), while *bla*_IMP-7_, *bla*_IMP-13_, and *bla*_IMP-26_ are mainly found in *P. aeruginosa* (5, 13). While *bla*_IMP-4_ is prevalent in both China and Australia, its spread is driven by IncN plasmids in China and IncC plasmids in Australia, *bla*_IMP-1_ is prevalent in Japan by IncN or IncF plasmids, indicating independent plasmid evolution pathways in different regions.

The dissemination of *bla*_IMP_-plasmids is influenced by factors like transfer-related elements, host species, geography, and plasmid size and structure (5, 6). Most plasmids have a complete conjugative modules including *oriT*, relaxase, T4CP and T4SS, all essential for conjugative transfer (34). Genomic analysis of pT117-2 identified a complete transfer region with *oriT*, MOB_F_-family relaxase gene, T4CP2 gene and T-type T4SS gene cluster, facilitating successful conjugation into *E. coli* J53.

A previous study identified IMP-encoding In*809*-like integrons within hybrid transposons of IncA/C2 plasmids from Australian wildlife and clinical *Enterobacteriaceae*, which featured conserved insertion sites but variable internal structures and resistance gene cassettes, thus demonstrating the structural variability, rapid evolution and genetic plasticity of IncC plasmids in mediating IMP dissemination (43). In line with the findings that diverse Inc plasmids (IncM, IncI1, IncF, etc.) drive IMP-4 spread via In*809*/In*1460* integrons through IS*26*- and Tn*1696*-associated mobilization in wildlife *Enterobacteriaceae* (44), our study further confirms that various Inc plasmids play a pivotal role in *bla*_IMP-4_ transmission across different *Klebsiella* species.

Unlike the typically genetic environment of IMP-encoding In*809*-like integrons in *Enterobacteriaceae*, the genetic context of *bla*_IMP-4_ on pT117-2 was highly conserved within the “IS*6100-mobC-ltrA-bla*_IMP-4_-*∆IntI1*-IS*26*” cassette. This structure is embedded in a class one integron (In*823*) with a truncated intl1 gene upstream. The presence of *bla*_IMP-4_ on In*823* highlights the role of integrons in the proliferation and dissemination of ARGs (45). The genetic context of *bla*_IMP-4_ on pT117-2 underscores the potential for horizontal gene transfer and the spread of ARGs, particularly through integrons, which have been recognized as significant contributors to the prevalence of ARGs.

While a previous study has delineated the prevalence, evolutionary dynamics, and genetic contexts of 61 *bla*_IMP_-harboring *Klebsiella* spp. strains isolated in China between 2016 and 2021 (6), the present study describes a novel sequence type ST of *K. variicola* with the *bla*_IMP-4_ on an IncN-type plasmid. Furthermore, we provide a comprehensive analysis of conjugative transfer elements in 123 *bla*_IMP_-harboring plasmids derived from *Klebsiella* spp. worldwide. These findings significantly supplement and extend our current understanding of the plasmid-mediated transmission mechanisms of the *bla*_IMP_ gene.

Our study also has limitations. We focused solely on plasmid evolution and composition in the *Klebsiella* spp., excluding non-Klebsiella *Enterobacteriaceae* and other Gram-negative bacilli. This narrow scope restricts our understanding of *bla*_IMP_ epidemiology. Additionally, we did not conduct extensive evolutionary and comparative analyses of chromosomal genomes. Future research should include broader bacterial groups and more in-depth chromosomal analysis to provide a comprehensive view of *bla*_IMP_ dissemination and resistance mechanisms.

### Conclusion

In this study, we identified a carbapenem-resistant strain of *K. variicola* T117, which harbored the *bla*_IMP-4_-carrying IncN conjugative plasmid pT117-2, with *bla*_IMP-4_ and *qnrS1* coexisting on the plasmid. Our analysis of 123 *bla*_IMP_-harboring plasmids from *Klebsiella* spp., including pT117-2, revealed nine variants, with *bla*_IMP-4_ and *bla*_IMP-1_ being the most prevalent. The *bla*_IMP-4_ gene was associated with IncN (50 kb, conjugative) and untypeable (300 kb, non-mobilizable) plasmids in China, IncC (200 kb) and IncM2 (80 kb) conjugative plasmids in Australia, while *bla*_IMP-1_ was linked to IncN (50 kb), IncM (80 kb), and IncFII (80–200 kb) conjugative plasmids in Japan. Comparative analyses showed that the *bla*_IMP-4_ gene is highly enriched in *K. pneumoniae*, predominantly transmitted horizontally via IncN-type plasmids, and geographically clustered in East Asia. The detection of *bla*_IMP-4_ in ST146 *K. variicola* underscores the threat of carbapenem-resistant gene dissemination, highlighting the need for ongoing vigilance and research to address its public health impact.

## Supplementary Material

Reviewer comments

## Data Availability

The genome sequence of *K. variicola* T117, which contained a chromosome and two plasmids, was submitted to GenBank under the accession numbers CP193999–CP194001.

## SUPPLEMENTAL MATERIAL

OPEN PEER REVIEW reviewer-comments.pdf An accounting of the reviewer comments and feedback.

ASM does not own the copyrights to Supplemental Material that may be linked to, or accessed through, an article. The authors have granted ASM a non-exclusive, world-wide license to publish the Supplemental Material files. Please contact the corresponding author directly for reuse.
