# Supplementary material for: Genomic characterization of blaIMP-harboring plasmids in Klebsiella spp
Source: Microbiol Spectr. 2026 Jul 2;14(8):e03790-25. doi: 10.1128/spectrum.03790-25 (PMC13436412; doi:10.1128/spectrum.03790-25)
Supplement: Supplementary file 1 [file reviewer-comments.pdf]

Microbiology Spectrum

Genomic Characterization of bla_{IMP}-Harboring Plasmids in *Klebsiella* spp.

Mingxiao chen, ting Deng, Yi Zhang, Yitong Han, Jie Li, ling Wang, Qiang Zhou, Xiaobin Li, Yu Gan, and Yuqing Weng

Corresponding Author(s): Mingxiao chen, Guangzhou Medical University

Review Timeline:

Submission Date:	November 29, 2025
Editorial Decision:	January 20, 2026
Revision Received:	March 12, 2026
Editorial Decision:	April 9, 2026
Revision Received:	April 15, 2026
Accepted:	April 19, 2026

Editor: Mor Lurie-Weinberger

Reviewer(s): Disclosure of reviewer identity is with reference to reviewer comments included in decision letter(s). The following individuals involved in review of your submission have agreed to reveal their identity: Yunsong Yu (Reviewer #1)

Transaction Report:

(Note: With the exception of the correction of typographical or spelling errors that could be a source of ambiguity, letters and reports are not edited. The original formatting of letters and referee reports may not be reflected in this compilation.)

DOI: https://doi.org/10.1128/spectrum.03790-25

Re: Spectrum03790-25 (**Genomic Characterization of *bla*$_{IMP}$-Harboring Plasmids in *Klebsiella* spp.**)

Dear Dr. xiao ming chen:

Thank you for the privilege of reviewing your work. Below you will find my comments, instructions from the Spectrum editorial office, and the reviewer comments.

Please return the manuscript within 60 days; if you cannot complete the modification within this time period, please contact me. If you do not wish to modify the manuscript and prefer to submit it to another journal, notify me immediately so that the manuscript may be formally withdrawn from consideration by Spectrum.

Revision Guidelines
To submit your modified manuscript, log into the submission site at https://spectrum.msubmit.net/cgi-bin/main.plex. Go to Author Tasks and click the appropriate manuscript title to begin. The information you entered when you first submitted the paper will be displayed; update this as necessary. Note the following requirements:

• Upload point-by-point responses to the issues raised by the reviewers in a file named "Response to Reviewers," NOT in your cover letter.
• Upload a compare copy of the manuscript (without figures) as a "Marked-Up Manuscript" file.
• Upload a clean .DOC/.DOCX version of the revised manuscript and remove the previous version.
• Each figure must be uploaded as a separate, editable, high-resolution file (TIFF or EPS preferred), and any multipanel figures must be assembled into one file.
• Any supplemental material intended for posting by ASM should be uploaded with their legends separate from the main manuscript. You can combine all supplemental material into one file (preferred) or split it into a maximum of 10 files with all associated legends included.

For complete guidelines on revision requirements, see our Submission and Review Process webpage. Submission of a paper that does not conform to guidelines may delay acceptance of your manuscript.

Data availability: ASM policy requires that data be available to the public upon online posting of the article, so please verify all links to sequence records, if present, and make sure that each number retrieves the full record of the data. If a new accession number is not linked or a link is broken, provide Spectrum production staff with the correct URL for the record. If the accession numbers for new data are not publicly accessible before the expected online posting of the article, publication may be delayed; please contact production staff (Spectrum@asmusa.org) immediately with the expected release date.

Publication Fees: For information on publication fees and which article types are subject to charges, visit our website. If your manuscript is accepted for publication and any fees apply, you will be contacted separately about payment during the production process; please follow the instructions in that e-mail. Arrangements for payment must be made before your article is published.

ASM Membership: Corresponding authors may join or renew ASM membership to obtain discounts on publication fees. Need to upgrade your membership level? Please contact Customer Service at Service@asmusa.org.

The ASM Journals program strives for constant improvement in our submission and publication process. Please tell us how we can improve your experience by taking this quick Author Survey.

Thank you for submitting your paper to Spectrum.

Sincerely,
Mor Lurie-Weinberger
Editor
Microbiology Spectrum

Reviewer #1 (Comments for the Author):

This study identified a carbapenem-resistant Klebsiella variicola isolate that harbours IMP-4, and subsequently analysis the characters of IMP plasmids in Klebsiella spp.
Line 81: You state that the research of IMP plasmid in carbapenem-resistant Klebsiella variicola is limited. Did you fully characterise the characteristic of IMP plasmid and close the knowledge gap? Since a similar study has been published from the same country (PMID: 38383740), could you use a short concluding paragraph to state explicitly what is novel about your own

study.
Line 168: Method 2.4 is confused to me. As you write that you compared IMP-4 sequence to nr database, but you don't explain how the entire plasmid were identified. And through the section of methodology, a lot of key parameters were missing.
Line 219: what is the difference between Table 1 and Table 2?
Line 146: PAE3 ?
Line 264: databae?

Reviewer #2 (Comments for the Author):

In the current study, the authors described the characterization of IMP-encoding plasmids from K. pneumoniae isolates. The study is well iorganised and presented.
Comments:
line 457: a previous study has shown the variabillity of IncC plasmids with In809 integrons (PMID: 28696228). Please discuss this study.
line 474: the impact of IMP-encoding plasmids in Enterobacteriaceae has been discussed in a previous study (PMID: 29483121). This point should e discussed in the text.

Dear Reviewers,

We sincerely appreciate your careful review and valuable comments on our manuscript. These suggestions have greatly helped us improve the quality and clarity of our work. We have carefully addressed each comment point by point, and the corresponding revisions have been made in the manuscript. The detailed responses are as follows:

Response to Reviewer #1

Comment 1 (Line 81): You state that the research of IMP plasmid in carbapenem-resistant Klebsiella variicola is limited. Did you fully characterise the characteristic of IMP plasmid and close the knowledge gap? Since a similar study has been published from the same country (PMID: 38383740), could you use a short concluding paragraph to state explicitly what is novel about your own study.

Response: We sincerely appreciate this critical comment. First, we confirm that we have fully characterized the bla_{IMP}-harboring plasmid in the carbapenem-resistant *Klebsiella* spp. isolate in this study, including plasmid replicon type, size, resistance gene cassette composition, conjugative transfer ability, and phylogenetic relationship with other bla_{IMP}-harboring plasmids. These comprehensive characterizations have effectively supplemented the current limited knowledge of bla_{IMP}-harboring plasmids in carbapenem-resistant *Klebsiella* spp., thereby narrowing the existing knowledge gap. Regarding the novelty of our study compared with the previously published similar study (PMID: 38383740) from the same country, we have added a dedicated concluding paragraph to explicitly clarify the innovations, which are as follows: (1) The study with PMID: 38383740 mainly investigated "strains" of *Klebsiella* spp., while the present study focuses on carbapenem-resistant "plasmids" of *Klebsiella* spp., filling the gap in the research on bla_{IMP}-harboring plasmids in carbapenem-resistant *Klebsiella* spp.. (2) We identified a novel IncN-type replicon in the $bla_{\mathrm{IMP-4}}$-harboring plasmid in ST164 *K. variicola*, which has not been reported in the study with PMID: 38383740. (3) Our study further explored the conjugative transfer capacity of the bla_{IMP}-harboring plasmids, providing more comprehensive data for evaluating the transmission risk of bla_{IMP}-carrying plasmids. The added concluding paragraph has been inserted at the end of the Discussion section (revised Lines 488-495 in the manuscript).

Comment 2 (Line 168): Method 2.4 is confused to me. As you write that you compared IMP-4 sequence to nr database, but you don't explain how the entire plasmid were identified. And through the section of methodology, a lot of key parameters were missing.

Response: We apologize for the confusion caused by the unclear description of Method 2.4. We have thoroughly revised this section to clarify the experimental process and supplement the missing key parameters. Specifically, we have added detailed steps for the identification of the entire plasmid: after obtaining the IMP-4 gene sequence, we used it as a seed sequence to perform local BLAST against the whole-genome sequencing (WGS) data of the target plasmids. The revised Method

2.4 and the supplemented parameters have been updated in the manuscript (revised Lines 170-175).

Comment 3 (Line 219): What is the difference between Table 1 and Table 2?
We apologize for the confusion caused by the incorrect original Table 2, which has now been revised and corrected. Table 1 details the molecular characteristics of the carbapenem-resistant *K.variicola* isolate (e.g., antimicrobial susceptibility profiles, resistance gene carriage), while Table 2 focuses on the core features of the *bl*a$_{IMP-4}$-harboring plasmid (revised Lines 719-720).

Comment 4 (Line 146): PAE3？
Response: This is a typographical error. The correct term should be " T117 strain" (the name of the target isolate). We have corrected this typo in the manuscript (Line 142) to ensure the accuracy of the isolate name(revised Line 146).

Comment 5 (Line 264): databae? Response: This is a spelling error. We have corrected "databae" to "database" in the manuscript(revised Line 257).

Response to Reviewer #2

Comment 1 (Line 457): A previous study has shown the variability of IncC plasmids with In809 integrons (PMID: 28696228). Please discuss this study.
Response: We appreciate this suggestion. We have added a discussion on the study with PMID: 28696228 in the Discussion section (revised Lines 465-470).

Comment 2 (Line 474): The impact of IMP-encoding plasmids in Enterobacteriaceae has been discussed in a previous study (PMID: 29483121). This point should be discussed in the text.
Response: Thank you for reminding us of this important study. We have supplemented the discussion on the study with PMID: 29483121 in the Discussion section (revised Lines 470-474).

As the data contained in the supplementary table S1 has been fully represented in the main figures, the table S1have been deemed unnecessary and removed. Accordingly, all citations to Table S1 in the main text have also been deleted.

Sincerely,
Qiang Zhou

Re: Spectrum03790-25R1 (**Genomic Characterization of *bla*$_{IMP}$-Harboring Plasmids in *Klebsiella* spp.**)

Dear Dr. Mingxiao chen:

Thank you for the privilege of reviewing your work. Below you will find instructions from the Spectrum editorial office, and the reviewer comments.

Dear author,

Please see the attached review regarding your revision. It appears to contain minor issues that need to be fixed before the paer can be accepted.

Please return the manuscript within 60 days; if you cannot complete the modification within this time period, please contact me. If you do not wish to modify the manuscript and prefer to submit it to another journal, notify me immediately so that the manuscript may be formally withdrawn from consideration by Spectrum.

Revision Guidelines
To submit your modified manuscript, log into the submission site at https://spectrum.msubmit.net/cgi-bin/main.plex. Go to Author Tasks and click the appropriate manuscript title to begin. The information you entered when you first submitted the paper will be displayed; update this as necessary. Note the following requirements:

• Upload point-by-point responses to the issues raised by the reviewers in a file named "Response to Reviewers," NOT in your cover letter.
• Upload a compare copy of the manuscript (without figures) as a "Marked-Up Manuscript" file.
• Upload a clean .DOC/.DOCX version of the revised manuscript and remove the previous version.
• Each figure must be uploaded as a separate, editable, high-resolution file (TIFF or EPS preferred), and any multipanel figures must be assembled into one file.
• Any supplemental material intended for posting by ASM should be uploaded with their legends separate from the main manuscript. You can combine all supplemental material into one file (preferred) or split it into a maximum of 10 files with all associated legends included.

For complete guidelines on revision requirements, see our Submission and Review Process webpage. Submission of a paper that does not conform to guidelines may delay acceptance of your manuscript.

Data availability: ASM policy requires that data be available to the public upon online posting of the article, so please verify all links to sequence records, if present, and make sure that each number retrieves the full record of the data. If a new accession number is not linked or a link is broken, provide Spectrum production staff with the correct URL for the record. If the accession numbers for new data are not publicly accessible before the expected online posting of the article, publication may be delayed; please contact production staff (Spectrum@asmusa.org) immediately with the expected release date.

Publication Fees: For information on publication fees and which article types are subject to charges, visit our website. If your manuscript is accepted for publication and any fees apply, you will be contacted separately about payment during the production process; please follow the instructions in that e-mail. Arrangements for payment must be made before your article is published.

ASM Membership: Corresponding authors may join or renew ASM membership to obtain discounts on publication fees. Need to upgrade your membership level? Please contact Customer Service at Service@asmusa.org.

The ASM Journals program strives for constant improvement in our submission and publication process. Please tell us how we can improve your experience by taking this quick Author Survey.

Thank you for submitting your paper to Spectrum.

Sincerely,
Mor Lurie-Weinberger
Editor
Microbiology Spectrum

Reviewer #3 (Comments for the Author):

This study isolated a clinical strain Klebsiella variicola T117 carrying the blaIMP-4 gene, and conducted a large-scale comparative genomics analysis by combining 123 plasmids carrying the blaIMP gene from the GenBank database. It systematically revealed the distribution characteristics of the blaIMP gene in different geographical regions and plasmid types. The study has a large amount of data and a systematic analysis, which holds significant public health importance. Although authors have revised the manuscript based on reviewers' comments, there are some deficiencies in data consistency, geographical expression standardization, and chart presentation, as follows:

1. The core content of the abstract and the conclusion are almost exactly the same, both repeating the main findings such as "blaIMP-4 is associated with IncN in China, and blaIMP-1 is associated with IncN/IncM/IncFII in Japan". The conclusion should avoid simply repeating the abstract and can include forward-looking implications for public health monitoring, the limitations of the current study, and future research directions, etc.
2. Line254. "antimicrobial resistance genes (ARGs)". This abbreviation has already been introduced at Line 218. Please either use the abbreviation "ARGs" consistently after its first definition, or remove the redefinition here; Line480 and Line483. The terms "antibiotic resistance genes" and "drug-resistant genes" should be written in the same way as before. It is recommended that the writing style of "antimicrobial resistance genes" throughout the text remain consistent. Moreover, the term "ARGs" can be used as an alternative when it appears later on.
3. 346-347. "We also analyzed the genetic environment of the blaIMP gene in the 36 IncP1-type plasmids from Klebsiella spp.." The descriptions before and after are inconsistent. Moreover, according to Figure 6D, there should be three IncP1 plasmids. The author is requested to verify and make the necessary corrections.
4. Line356. "two from Taiwan", Taiwan is an inalienable part of China. Please revise to reflect this, e.g., "two from Taiwan, China" or integrate Taiwan into the data for China. Do not list Taiwan separately alongside sovereign states.
5. Line376. "TniQ" should be written as: TniQ; Line471. "In809/In1460" should be written as: In809/In1460.
6. Line401-405. The previous paragraph stated that Clade VIII belongs to a conjugative plasmid, but the last paragraph said that this plasmid does not have oriT. These descriptions are contradictory. A plasmid without oriT cannot possess the typical conjugative transfer ability. Please recheck the oriTfinder analysis results. If it is indeed without oriT, Clade VIII should be reclassified as "non-mobilizable" or "mobilizable". If oriT actually exists, please correct the main text description.
7. Table 1. The first letters of the ARGs OqxB and OqxA should be in lowercase; "None", "-" refers to what? Table 2. The full name should be written when the term "VF" first appears. Table 3. Please explain in the table what R, I, and S specifically refer to; and what SDD refers to.
8. In Figure 1, the gel electrophoresis diagram lacks clear labels. On the left side of the figure, the molecular weights of each band of the Marker are indicated (such as 500 bp, 1000 bp).
9. Figure 2, Figure 3, Figure 4. Taiwan, as a part of China, cannot be listed separately and placed on par with China. The author is requested to make the necessary corrections.
10. Figure 6. The text descriptions in the figure are not clear enough. Please enhance the clarity.

Dear Reviewers,

We sincerely appreciate your careful review and valuable comments on our manuscript. These suggestions have greatly helped us improve the quality and clarity of our work. We have carefully addressed each comment point by point, and the corresponding revisions have been made in the manuscript. The detailed responses are as follows:

Response to Reviewer #1

Comment 1 (Line 81): You state that the research of IMP plasmid in carbapenem-resistant Klebsiella variicola is limited. Did you fully characterise the characteristic of IMP plasmid and close the knowledge gap? Since a similar study has been published from the same country (PMID: 38383740), could you use a short concluding paragraph to state explicitly what is novel about your own study.

Response: We sincerely appreciate this critical comment. First, we confirm that we have fully characterized the *bla*$_{IMP}$-harboring plasmid in the carbapenem-resistant *Klebsiella* spp. isolate in this study, including plasmid replicon type, size, resistance gene cassette composition, conjugative transfer ability, and phylogenetic relationship with other *bla*$_{IMP}$-harboring plasmids. These comprehensive characterizations have effectively supplemented the current limited knowledge of *bla*$_{IMP}$-harboring plasmids in carbapenem-resistant *Klebsiella* spp., thereby narrowing the existing knowledge gap. Regarding the novelty of our study compared with the previously published similar study (PMID: 38383740) from the same country, we have added a dedicated concluding paragraph to explicitly clarify the innovations, which are as follows: (1) The study with PMID: 38383740 mainly investigated "strains" of *Klebsiella* spp., while the present study focuses on carbapenem-resistant "plasmids" of *Klebsiella* spp., filling the gap in the research on *bla*$_{IMP}$-harboring plasmids in carbapenem-resistant *Klebsiella* spp.. (2) We identified a novel IncN-type replicon in the *bla*$_{IMP-4}$-harboring plasmid in ST164 *K. variicola*, which has not been reported in the study with PMID: 38383740. (3) Our study further explored the conjugative transfer capacity of the *bla*$_{IMP}$-harboring plasmids, providing more comprehensive data for evaluating the transmission risk of *bla*$_{IMP}$-carrying plasmids. The added concluding paragraph has been inserted at the end of the Discussion section (revised Lines 488-495 in the manuscript).

Comment 2 (Line 168): Method 2.4 is confused to me. As you write that you compared IMP-4 sequence to nr database, but you don't explain how the entire plasmid were identified. And through the section of methodology, a lot of key parameters were missing.

Response: We apologize for the confusion caused by the unclear description of Method 2.4. We have thoroughly revised this section to clarify the experimental process and supplement the missing key parameters. Specifically, we have added detailed steps for the identification of the entire plasmid: after obtaining the IMP-4 gene sequence, we used it as a seed sequence to perform local BLAST against the whole-genome sequencing (WGS) data of the target plasmids. The revised Method

2.4 and the supplemented parameters have been updated in the manuscript (revised Lines 170-175).

Comment 3 (Line 219): What is the difference between Table 1 and Table 2?
We apologize for the confusion caused by the incorrect original Table 2, which has now been revised and corrected. Table 1 details the molecular characteristics of the carbapenem-resistant *K.variicola* isolate (e.g., antimicrobial susceptibility profiles, resistance gene carriage), while Table 2 focuses on the core features of the *bl*a$_{IMP-4}$-harboring plasmid (revised Lines 719-720).

Comment 4 (Line 146): PAE3？
Response: This is a typographical error. The correct term should be " T117 strain" (the name of the target isolate). We have corrected this typo in the manuscript (Line 142) to ensure the accuracy of the isolate name(revised Line 146).

Comment 5 (Line 264): databae? Response: This is a spelling error. We have corrected "databae" to "database" in the manuscript(revised Line 257).

Response to Reviewer #2

Comment 1 (Line 457): A previous study has shown the variability of IncC plasmids with In809 integrons (PMID: 28696228). Please discuss this study.
Response: We appreciate this suggestion. We have added a discussion on the study with PMID: 28696228 in the Discussion section (revised Lines 465-470).

Comment 2 (Line 474): The impact of IMP-encoding plasmids in Enterobacteriaceae has been discussed in a previous study (PMID: 29483121). This point should be discussed in the text.
Response: Thank you for reminding us of this important study. We have supplemented the discussion on the study with PMID: 29483121 in the Discussion section (revised Lines 470-474).

As the data contained in the supplementary table S1 has been fully represented in the main figures, the table S1have been deemed unnecessary and removed. Accordingly, all citations to Table S1 in the main text have also been deleted.

Sincerely,
Qiang Zhou

Reviewer #3 (Comments for the Author):

This study isolated a clinical strain *Klebsiella variicola* T117 carrying the bla_{IMP-4} gene, and conducted a large-scale comparative genomics analysis by combining 123 plasmids carrying the bla_{IMP} gene from the GenBank database. It systematically revealed the distribution characteristics of the bla_{IMP} gene in different geographical regions and plasmid types. The study has a large amount of data and a systematic analysis, which holds significant public health importance. Although authors have revised the manuscript based on reviewers' comments, there are some deficiencies in data consistency, geographical expression standardization, and chart presentation, as follows:

1. The core content of the abstract and the conclusion are almost exactly the same, both repeating the main findings such as "bla_{IMP-4} is associated with IncN in China, and bla_{IMP-1} is associated with IncN/IncM/IncFII in Japan". The conclusion should avoid simply repeating the abstract and can include forward-looking implications for public health monitoring, the limitations of the current study, and future research directions, etc.

Response:

We sincerely thank the reviewer for the valuable comment. We have thoroughly revised the Conclusion section to avoid repetitive content with the Abstract. The revised version no longer simply duplicates core findings, and has supplemented public health implications, research limitations, as well as future research perspectives to enrich and deepen the conclusion.

2. Line254. "antimicrobial resistance genes (ARGs)". This abbreviation has already been introduced at Line 218. Please either use the abbreviation "ARGs" consistently after its first definition, or remove the redefinition here; Line480 and Line483. The terms "antibiotic resistance genes" and "drug-resistant genes" should be written in the same way as before. It is recommended that the writing style of "antimicrobial resistance genes" throughout the text remain consistent. Moreover, the term "ARGs" can be used as an alternative when it appears later on.

Response:

We thank the reviewer for the careful comment. We have carefully revised the full text to ensure consistent terminology and abbreviation usage for antimicrobial resistance genes (ARGs). Redundant redefinition at Line 254 has been removed, and expressions at Lines 480 and 483 have been unified accordingly.

3. 346-347. "We also analyzed the genetic environment of the bla_{IMP} gene in the 36 IncP1-type plasmids from *Klebsiella* spp.." The descriptions before and after are inconsistent. Moreover, according to Figure 6D, there should be three IncP1 plasmids. The author is requested to verify and make the necessary corrections.

Response:

We apologize for the numerical inconsistency caused by our carelessness. We have carefully verified the relevant content and corrected the number of IncP1 plasmids in the description to ensure consistency with Figure 6D.

4. Line356. "two from Taiwan", Taiwan is an inalienable part of China. Please revise to reflect this, e.g., "two from Taiwan, China" or integrate Taiwan into the data for China. Do not list Taiwan separately alongside sovereign states.

Respnse:

We sincerely appreciate the reviewer's important reminder. We firmly uphold the one-China principle and acknowledge that Taiwan is an inalienable part of China.In the main text and Figure 2, the region has been revised to "Taiwan, China". In Figures 3 and 4, Taiwan, together with Hong Kong and Macao Special Administrative Regions, has been clearly labeled as regions of China to ensure consistency with the one-China principle. All relevant descriptions have been corrected accordingly.

5. Line376. "TniQ" should be written as: TniQ; Line471. "In809/In1460" should be written as: In809/In1460.

Response:

We appreciate the reviewer's careful correction. The formatting of *TniQ* (Line 376) and In*809*/In*1460* (Line 471) has been revised accordingly, and consistent notation has been applied throughout the manuscript.

6. Line401-405. The previous paragraph stated that Clade VIII belongs to a conjugative plasmid, but the last paragraph said that this plasmid does not have oriT. These descriptions are contradictory. A plasmid without oriT cannot possess the typical conjugative transfer ability. Please recheck the oriTfinder analysis results. If it is indeed without oriT, Clade VIII should be reclassified as "non-mobilizable" or "mobilizable". If oriT actually exists, please correct the main text description.

Response :

We sincerely appreciate the reviewer's careful reading and valuable suggestion regarding the conjugative property of the plasmid in Clade VIII (Lines 401–405). We have re-examined the oriTfinder results and agree that no typical *oriT* was annotated in the initial analysis.

However, we would like to clarify that *oriT* sequences are generally short and highly variable. Meanwhile, oriTfinder uses strict thresholds of 80% nucleotide identity and 80% coverage for prediction, and some authentic oriT sequences are not included in its reference database. Due to these limitations, the prediction of oriT by oriTfinder is not always accurate or comprehensive.

In our comprehensive analysis, this plasmid carries a complete set of core genes required for conjugative transfer, including genes encoding relaxase, type IV coupling protein, and type IV secretion system components. **Therefore, we still classify the plasmid in Clade VIII as a conjugative plasmids**, and we have revised the relevant description in the manuscript to avoid ambiguity.

7. Table 1. The first letters of the ARGs OqxB and OqxA should be in lowercase; "None", "-" refers to what? Table 2. The full name should be written when the term "VF" first appears. Table 3. Please explain in the table what R, I, and S specifically refer to; and what SDD refers to.

Response :

We appreciate the reviewer's valuable suggestions on Tables 1, 2, and 3. All issues have been addressed, with details as follows:

Table 1: Revised to lowercase and italicized as *oqxB* and *oqxA* (standard ARG nomenclature), consistent throughout the manuscript. Added a footnote: "None" no antimicrobial resistance genes; "-"unidentified oriTs (undetected due to oriTfinder limitations).

Table 2: Since virulence was not the focus of this study, the "VF"related content has been deleted from Table 2.

Table 3: Added a footnote: "S = susceptible, I = intermediate, R = resistant, SDD = susceptible dose-dependent."

8. In Figure 1, the gel electrophoresis diagram lacks clear labels. On the left side of the figure, the molecular weights of each band of the Marker are indicated (such as 500 bp, 1000 bp).

Response:

We thank the reviewer for the constructive suggestion. The required molecular weight labels for each band of the Marker have been added on the left side of Figure 1 to improve the clarity of the gel electrophoresis image.

9. Figure 2, Figure 3, Figure 4. Taiwan, as a part of China, cannot be listed separately and placed on par with China. The author is requested to make the necessary corrections.

Response:

We sincerely appreciate the reviewer's important reminder. We firmly uphold the one-China principle and acknowledge that Taiwan is an inalienable part of China. We have revised the geographic labeling in Figure 2 accordingly. In Figures 3 and 4, Taiwan, Hong Kong, and Macao have been clearly labeled as regions of China to maintain consistency with the one-China principle.

10. Figure 6. The text descriptions in the figure are not clear enough. Please enhance the clarity.

Response:

We appreciate the reviewer's suggestion. The legends in Figure 6 have been enlarged to improve overall clarity and readability.

Re: Spectrum03790-25R2 (**Genomic Characterization of *bla*$_{IMP}$-Harboring Plasmids in *Klebsiella* spp.**)

Dear Dr. Mingxiao chen:

Your manuscript has been accepted, and I am forwarding it to the ASM production staff for publication. Your paper will first be checked to make sure all elements meet the technical requirements. ASM staff will contact you if anything needs to be revised before copyediting and production can begin. Otherwise, you will be notified when your proofs are ready to be viewed.

Data Availability: ASM policy requires that data be available to the public upon online posting of the article, so please verify all links to sequence records, if present, and make sure that each number retrieves the full record of the data. If a new accession number is not linked or a link is broken, provide production staff with the correct URL for the record. If the accession numbers for new data are not publicly accessible before the expected online posting of the article, publication may be delayed; please contact ASM production staff immediately with the expected release date.

Publication Fees: For information on publication fees and which article types have charges, please visit our website. We have partnered with Copyright Clearance Center (CCC) to collect author charges. If fees apply to your paper, you will receive a message from no-reply@copyright.com with further instructions. For questions related to paying charges through RightsLink, please contact CCC at ASM_Support@copyright.com or toll free at +1-877-622-5543. CCC makes every attempt to respond to all emails within 24 hours.

ASM Membership: Corresponding authors may join or renew ASM membership to obtain discounts on publication fees. Need to upgrade your membership level? Please contact Customer Service at Service@asmusa.org.

Embargo Policy: A press release may be issued as soon as the manuscript is posted on the Spectrum Latest Articles webpage. The corresponding author will receive an email with the subject line "ASM Journals Author Services Notification" when the article is available online.

The ASM Journals program strives for constant improvement in our submission and publication process. Please tell us how we can improve your experience by taking this quick Author Survey.

Thank you for submitting your paper to Spectrum.

Sincerely,
Mor Lurie-Weinberger
Editor
Microbiology Spectrum
